# Entropy Analysis and Image Encryption Application Based on a New Chaotic System Crossing a Cylinder

**DOI:** 10.3390/e21100958

**Published:** 2019-09-30

**Authors:** Alaa Kadhim Farhan, Nadia M.G. Al-Saidi, Abeer Tariq Maolood, Fahimeh Nazarimehr, Iqtadar Hussain

**Affiliations:** 1 Department of Computer Sciences, University of Technology, Baghdad 10011, Iraq; 2 Department of Applied Sciences, University of Technology, Baghdad 10011, Iraq; nadiamg08@gmail.com; 3 Department of Biomedical Engineering, Amirkabir University of Technology, 350 Hafez Ave., Tehran 1591634311, Iran; fahimenazarimehr@yahoo.com; 4 Department of Mathematics, Statistics and Physics, Qatar University, Doha 2713, Qatar

**Keywords:** chaotic flow, cylinder, bifurcation, multistability, complexity, encryption

## Abstract

Designing chaotic systems with specific features is a hot topic in nonlinear dynamics. In this study, a novel chaotic system is presented with a unique feature of crossing inside and outside of a cylinder repeatedly. This new system is thoroughly analyzed by the help of the bifurcation diagram, Lyapunov exponents’ spectrum, and entropy measurement. Bifurcation analysis of the proposed system with two initiation methods reveals its multistability. As an engineering application, the system’s efficiency is tested in image encryption. The complexity of the chaotic attractor of the proposed system makes it a proper choice for encryption. States of the chaotic attractor are used to shuffle the rows and columns of the image, and then the shuffled image is XORed with the states of chaotic attractor. The unpredictability of the chaotic attractor makes the encryption method very safe. The performance of the encryption method is analyzed using the histogram, correlation coefficient, Shannon entropy, and encryption quality. The results show that the encryption method using the proposed chaotic system has reliable performance.

## 1. Introduction

The chaotic dynamic has been a hot topic recently [1,2,3]. For many years, there was a hypothesis that chaotic attractors are related to a saddle point equilibria [4,5]. In the last decade, some chaotic systems were proposed, which were counterexamples of that hypothesis [6,7]. Chaotic systems with stable equilibria [8], chaotic flows with no equilibria [9,10], and chaotic flow with circular equilibria [11,12] are some of such counterexamples. Investigating new chaotic flows has been done in the literature [13]. Many chaotic flows with various features are being proposed every year [14,15,16]. Researchers hope that such studies help to investigate the reason of generating chaotic attractors [17,18,19,20]. Dynamical properties of two discs with viscous friction and multiple delays have been studied in [21]. An infinite lattice of chaotic attractors was discussed in [22]. Chaotic dynamics can be modeled using electronic circuits [23,24]. 

Each attractor of a system has a basin of attraction [25]. Based on the basin of attraction, attractors can be categorized into self-excited or hidden attractors [26,27]. An attractor is self-excited if its basin of attraction has an intersect with unstable equilibrium, while it is hidden if it does not [26,28]. Hidden attractors of an economical supply have been studied in [29]. In [30], hidden attractors of Rabinovich-Fabrikant system have been investigated. Multistability is an exciting phenomenon in the study of dynamical systems [31,32,33]. Many studies have been done on chaotic systems with multistability [34]. In the multistable systems, each attractor has its basin of attraction [35]. 

There are some useful measurements to quantify chaotic dynamics. Lyapunov exponent is one of the most reliable measures in this area [36]. In the chaotic attractors, there is at least one positive Lyapunov exponent [37]. Entropy is another measurement which can be used in the study of chaotic attractors [38]. Different entropy measures have been proposed to study the complexity of chaotic attractors [39]. Chaotic dynamics and their complexities have been analyzed in [40,41,42]. Local entropy has been used for image segmentation in [43]. 

In these days, the transmission of data without leakage is an important topic. Many methods have been proposed for data encryption [44,45,46]. Chaotic systems have many applications in various areas, such as biology and communication [47,48,49]. In [50], chaotic dynamics were investigated in the cryptocurrency market. Some features of chaotic dynamics, such as randomness and sensitivity to initial conditions, are desirable in the field of cryptology [51,52]. The unpredictability of chaotic time-series is an important feature, which is useful in the encryption [53]. Many studies have been done on the application of chaotic systems in encryption [54,55]. In [56], a watermarking method to embed an invisible watermark into the intra-frames of a MPEG video sequence has been studied. A hybrid non-blind MPEG video watermarking method was proposed in [57]. A robust image watermarking method, such as copyright protection, was studied in [58]. 

In this paper, a new chaotic system is proposed. The system has a special property which crosses a pre-defined cylinder repeatedly. The system is introduced in Section 2. The structural features of the proposed system are analyzed in Section 3. Additionally, bifurcation diagram, Lyapunov exponents, and entropy analysis of the system are studied in Section 3. In Section 4, an encryption method based on the proposed system is presented, and its results are discussed. Section 5 is the conclusion.

## 2. The New Chaotic System and Its Structural Properties

The novel three-dimensional chaotic system is as follows:

(1) $$\begin{array}{c} \dot{x}=\;z\\ \dot{y}=x^{2}+y^{2}-a^{2}\\ \dot{z}=\;0.4z+3xy. \end{array}$$

**Figure 1:**
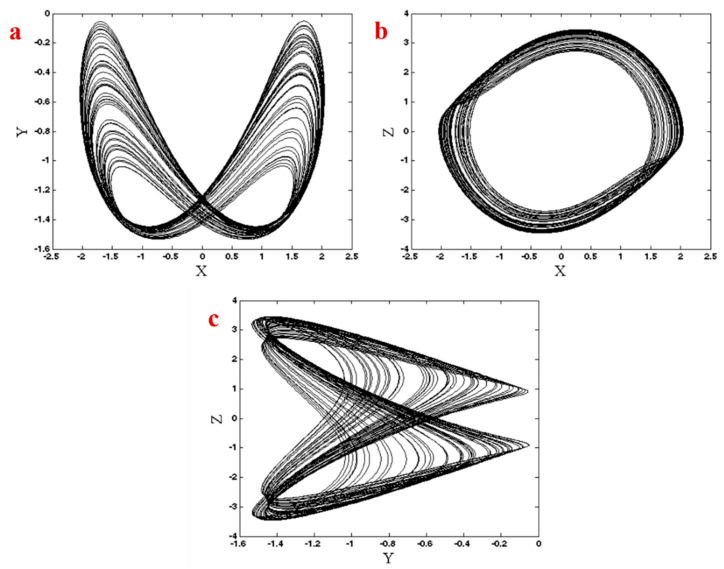
Chaotic attractor of System (1) in three different planes, (**a**) x−y plane, (**b**) x−z plane, (**c**) y−z plane.

The system shows chaotic dynamic in a = 1.7 with initial conditions $\left(x_{0},y_{0},z_{0}\right)\;=\;\left(0.29,-1.81,0.17\right)$. Figure 1 shows the chaotic attractor in three different projections. 

## 3. Dynamical Properties of the Proposed System

In the study of dynamical systems, bifurcation diagram is handy. It shows various dynamics of the system by changing parameters. In the following parts of this section, dynamical properties of the proposed system have been discussed by changing parameters.

### 3.1. Equilibrium Points and Their Stability

To investigate equilibrium points of the system, the right-hand side of Equation (1) should be zero as follows:

(2) $$\begin{array}{c} z\;=\;0\\ x^{2}+y^{2}-a^{2}\;=\;0.\\ xy\;=\;0 \end{array}$$

**Figure 2:**
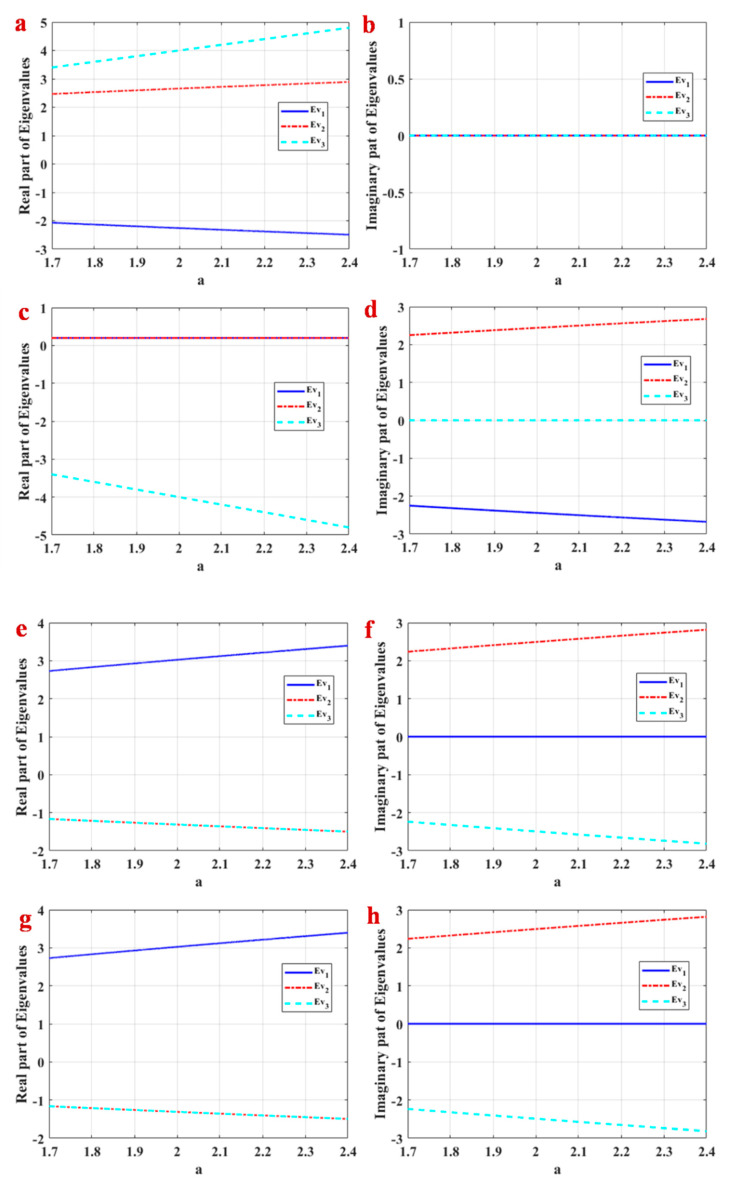
Real and imaginary parts of equilibrium points in a∈[1.7,2.4]. (**a**) Real part of Eigenvalues of $Eq_{1}$, (**b**) imaginary part of Eigenvalues of $Eq_{1}$, (**c**) real part of Eigenvalues of $Eq_{2}$, (**d**) imaginary part of Eigenvalues of $Eq_{2}$, (**e**) real part of Eigenvalues of $Eq_{3}$, (**f**) imaginary part of Eigenvalues of $Eq_{3}$, (**g**) real part of Eigenvalues of $Eq_{4}$, (**h**) imaginary part of Eigenvalues of $Eq_{4}$.

Therefore, we have four equilibrium points as $Eq_{1}:\;\left(0,a,0\right),\;Eq_{2}:\;\left(0,-a,0\right),\;Eq_{3}:\;\left(a,0,0\right),\;Eq_{4}:\;\left(-a,0,0\right)$. The stability of these equilibrium points is investigated using the Jacobian matrix and roots of characteristic equations in each equilibrium point. Figure 2 shows the real and imaginary parts of equilibrium points for a∈[1.7,2.4]. Figure 2a,b shows Eigenvalues of $Eq_{1}$. $Eq_{1}$ is a saddle point in the studied interval, and it is not spiral since the imaginary part of Eigenvalues is zero. Figure 2c,d shows Eigenvalues of $Eq_{2}$. It shows that $Eq_{2}$ has two complex conjugates with a positive real part and one negative real Eigenvalue. Therefore, it is a spiral saddle point. Eigenvalues of $Eq_{3}$ and $Eq_{4}$ are shown in Figure 2e–h. It shows that these equilibrium points are saddles and spiral when there are one positive and two negative real parts of Eigenvalues. Numerical investigations show that initial conditions in a small neighborhood of equilibrium points lead to the chaotic attractor, so the attractor is self-excited. 

### 3.2. Attractor around a Pre-Defined Cylinder

**Figure 3:**
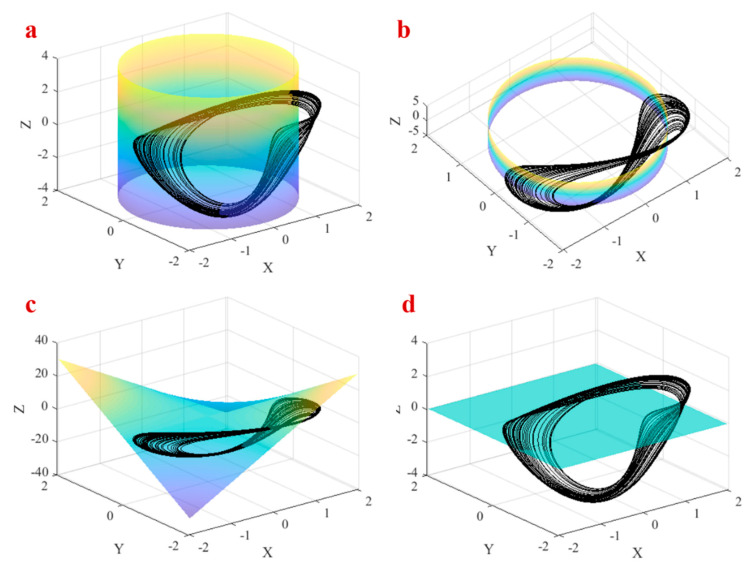
The chaotic attractor in a = 1.7 and (**a**) the condition $\langle x^{2}+y^{2}\rangle \;=a^{2}$, (**b**) the condition $\langle x^{2}+y^{2}\rangle \;=a^{2}$ from another viewpoint, (**c**) the condition 〈0.4z+3xy〉 = 0, (**d**) the condition 〈z〉 = 0.

The average of $\dot{x},\dot{y}$, and $\dot{z}$ of the system should be zero to have a bounded solution [59]. Therefore, in each attractor of the system, such as periodic and chaotic, the average of each derivative is zero. In other words, we have $\langle \dot{x}\rangle \;=\;\langle \dot{y}\rangle \;=\;\langle \dot{z}\rangle \;=\;0$. Thus, 〈z〉 = 0, $\langle x^{2}+y^{2}\rangle \;=a^{2}$, and 〈0.4z+3xy〉 = 0. The condition 〈z〉 = 0 means that the attractor of the system should be above the plane z=0 at sometimes and bellow the plane at some other times. Therefore 〈z〉 can be zero on the attractor of the proposed system. Condition $\langle x^{2}+y^{2}\rangle \;=a^{2}$ means that each bounded solution of the proposed system should cross the inside and outside of the cylinder $x^{2}+y^{2}\;=\;a^{2}$ repeatedly. The attractor of the system should cross the manifold z=−7.5xy to satisfy the condition 〈0.4z+3xy〉 = 0. Figure 3 shows the chaotic attractor in a = 1.7 and these three conditions which are satisfied by the attractor. Figure 3a,b shows the cylinder $x^{2}+y^{2}\;=1.7^{2}$ (from two points of view) which is crossed by the chaotic attractor repeatedly. The manifold z = −7.5xy and the chaotic attractor are shown in Figure 3c of the figure. Finally, the plane z = 0 and the attractor are shown in Figure 3d. 

### 3.3. Bifurcation Diagram

**Figure 4:**
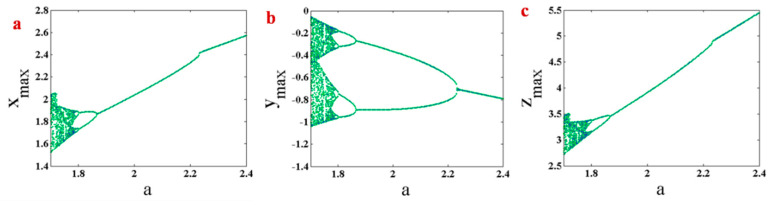
Bifurcation diagram of System (1) with backward continuation and the first initial conditions $\left(x_{0},y_{0},z_{0}\right)\;=\;\left(0.29,-1.81,0.17\right)$. (**a**) maximum values of x variable by changing parameter a; (**b**) maximum values of y variable by changing parameter a; (**c**) maximum values of z variable by changing parameter a.

**Figure 5:**
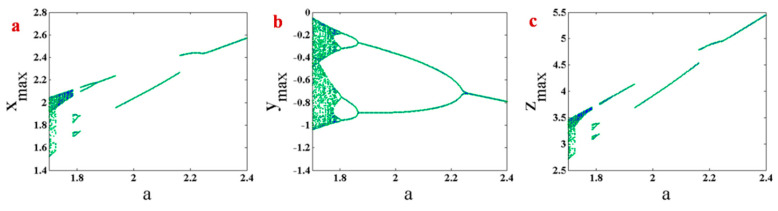
Bifurcation diagram of System (1) with constant initial conditions $\left(x_{0},y_{0},z_{0}\right)\;=\;\left(0.29,-1.81,0.17\right)$. (**a**) maximum values of x variable by changing parameter a; (**b**) maximum values of y variable by changing parameter a; (**c**) maximum values of z variable by changing parameter a.

Here, the bifurcation diagram of System (1) with respect to changing parameter a is studied. Bifurcation diagram can be plotted by different initiation methods. Two methods are used in this paper. The first method is backward continuation. In this method, the parameter is decreasing, and initial conditions in each parameter are selected from the end values of the states in the previous parameter, which is higher than the current parameter. The second method is plotting bifurcation diagram with constant initial conditions. Figure 4 shows a bifurcation diagram of System (1) with backward continuation. Initial conditions in the first parameter (a=2.4) are $\left(x_{0},y_{0},z_{0}\right)=\left(0.29,-1.81,0.17\right)$. The system has a period-doubling route to chaos by decreasing parameter a. Bifurcation diagram of System (1) with constant initial conditions $\left(x_{0},y_{0},z_{0}\right)=\left(0.29,-1.81,0.17\right)$ are plotted in Figure 5. The figure shows a period-doubling route to chaos by decreasing parameter a. However, some jumps can be seen in this route. Comparing bifurcation diagrams which are plotted by two initiation methods in Figure 4 and Figure 5, it can be seen that the system has multistability since its attractor can differ by varying initial conditions.

### 3.4. Lyapunov Exponents

**Figure 6:**
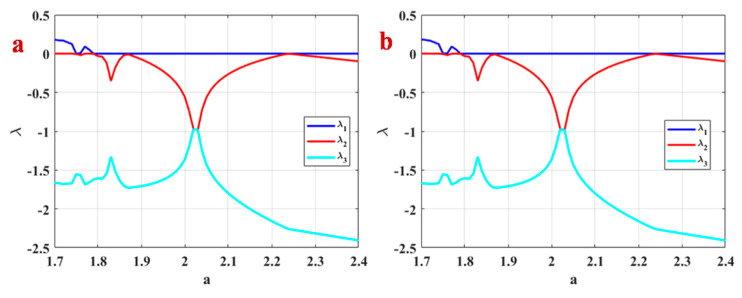
Lyapunov exponents of System (1) (**a**) with backward continuation, (**b**) with constant initial conditions.

Lyapunov exponents of System (1) related to the bifurcation diagrams of Figure 4 and Figure 5 are calculated using Wolf’s method [60] and run time 20000. Figure 6a presents Lyapunov exponents of System (1) with backward continuation. Positive Lyapunov exponents of the system in some intervals of parameter a prove the existence of chaos. Figure 6b shows Lyapunov exponents of System (1) with constant initial conditions. Comparing Lyapunov exponents plotting by these two methods shows that however, the system shows multistability by two initiation methods, but the quality of the multistable attractors is the same since their Lyapunov exponents are the same.

### 3.5. Entropy Analysis

Entropy is a measure of complexity. Entropy can be used in the analysis of biological signals [61,62]. A well-known entropy is Shannon entropy [63], as shown in Equation (3). In this equation, $\rho_{i}$ is the probability of each possible i state.

(3) $$H\;=\;-\underset{i}{\sum }\rho_{i}\log\left(\rho_{i}\right)$$

Another entropy measure which is more applicable in chaotic systems is Kolmogorov–Sinai entropy as Equation (4). $\tau_{i}$ is the first Poincaré recurrence times (FPRs). β is a D-dimensional box in the state space with side ε, and the FPRs are observed. ρ(τ,β) is the probability distribution of $\tau_{i}$. This entropy is positive in chaotic dynamics [64,65]. Moreover, approaching the bifurcation points can be seen in the entropy if it is calculated without removing transient time. It is because of the slowness near bifurcation points, which causes the state to be more distributed.

(4) $$H_{ks}\left(\beta \left[\varepsilon \right]\right)=\frac{1}{\tau_{min}\left(\beta \left[\varepsilon \right]\right)\;}\underset{\tau }{\sum }\rho \left(\tau ,\beta \left[\varepsilon \right]\right)\log\left(\frac{1}{\rho \left(\tau ,\beta \left[\varepsilon \right]\right)}\right)\;$$

**Figure 7:**
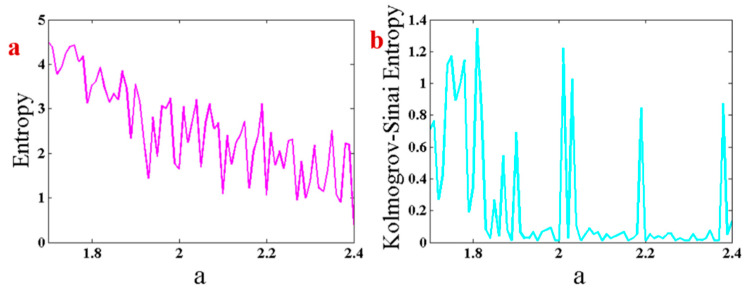
(**a**) Shannon entropy and (**b**) Kolmogorov–Sinai entropy of the proposed system with respect to changing parameter a and backward continuation method.

Figure 7a,b shows the results of Shannon entropy and Kolmogorov–Sinai entropy of the proposed system with respect to changing parameter a and backward continuation method. By increasing parameter a an inverse route of a period-doubling route to chaos happens. Thus, the entropy is decreased by increasing parameter a. Kolmogorov–Sinai entropy shows positive values in chaotic regions and also when approaching bifurcation points. 

**Figure 8:**
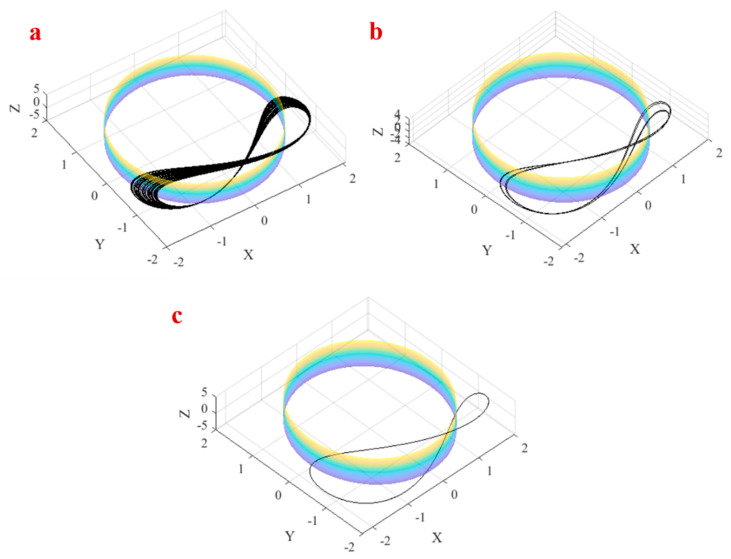
The attractor of System (1) in (**a**) a=1.74, (**b**) a=1.8, (**c**) a=2.

Investigating variations of the attractor of the system by changing parameter a is very interesting. Parameter a changes the radios of the cylinder. It was discussed that each attractor of the system should cross the cylinder repeatedly. Therefore, we can change the domain of attractor in the x−y plane by changing parameter a. Figure 7 shows the attractor of System (1) which crosses the cylinder with radios a in three parameters  = 1.74,a = 1.8, a = 2 , as shown in Figure 8a–c. 

The system is highly dependent on parameter a since it can have various dynamics in a small interval. Moreover, initial conditions are critical in the dynamic of the system since the system is multistable. In overall, the sensitivity of the dynamic of the system to the parameter and initial conditions makes it a proper choice for encryption.

## 4. Image Encryption

The proposed system has a complex chaotic dynamic. Besides the sensitivity of its chaotic attractor to initial conditions, the system is multistable, which makes it more complex. The encryption method, which is based on [55] is discussed below. 

### 4.1. Encryption Method

The parameter a and initial conditions of the chaotic attractor of Figure 1 enter the first block of the Algorithm 1. The system is run using the Rung–Kutta method (Ode15s in Matlab program) with constant time steps 0.01 and run time 300. Therefore, 30000 bits are generated using the time series of each variable. The float values of the time series are transferred to 32-bit binary values with 3 bits for the integer part and 29 bits for fraction part since the maximum amplitude of the chaotic attractor 4. In the next step, the 20 least significant bits are selected to be used in the generation of random numbers. Then the 20 least significant bits of the following values of time series are put in a vector which is used in the encryption process. 

**Figure 9:**
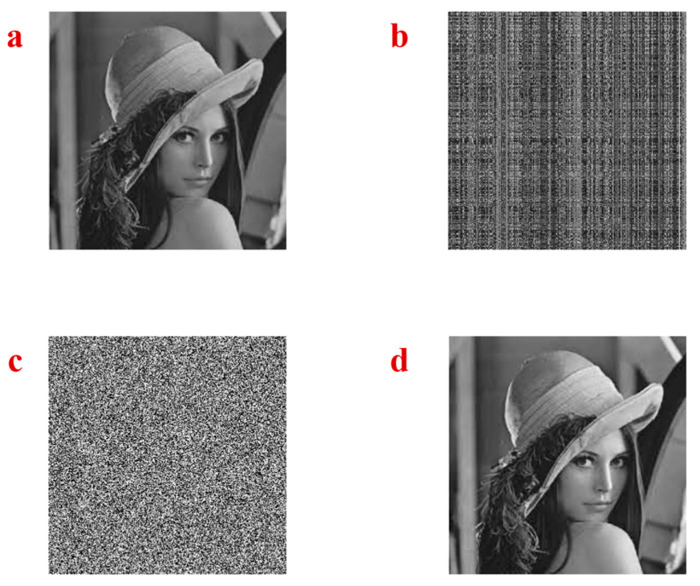
(**a**) The original Lena image, (**b**) the shuffled rows and columns of the Lena image, (**c**) the encrypted image, (**d**) the decrypted image.

Lena image is used to test the power of the chaotic attractor of the proposed system in image encryption. The image is shown in Figure 9a. In the first step of encryption, the Lena image is loaded with the size 256×256. Then a random line mixing is used to shuffle rows and columns of the picture. The random vector of x variable, which was generated in the previous part, is used to shuffle the rows of the image. Every eight bits of the vector are considered as a row number. In this step, it is possible that some redundancies exist in the generated indices. Therefore, we remove the duplicates until 256 distinct numbers between 1 to 256 are generated. After that, the rows are shuffled using the random indices. Then, the random vector of z variable is used to shuffle the columns of the image with the same process. Figure 9b shows the shuffled rows and columns of the Lena image. Then, the shuffled image is converted to a vector. In the next step, the image is converted to binary values with eight bits for the integer part, and its values are XORed with the eight bits sequences of the random generated sequence of x and y variables. After that, the results are converted to decimal. Next, the encrypted vector is converted into the encrypted image which is shown in Figure 9c. The parameter and initial conditions of System (1) have been sent to the receiver side as the key in the Algorithm 1. In the decryption, the inverse of the encryption steps should be used to obtain the original image, as shown in Figure 9d.

**Figure 10:**
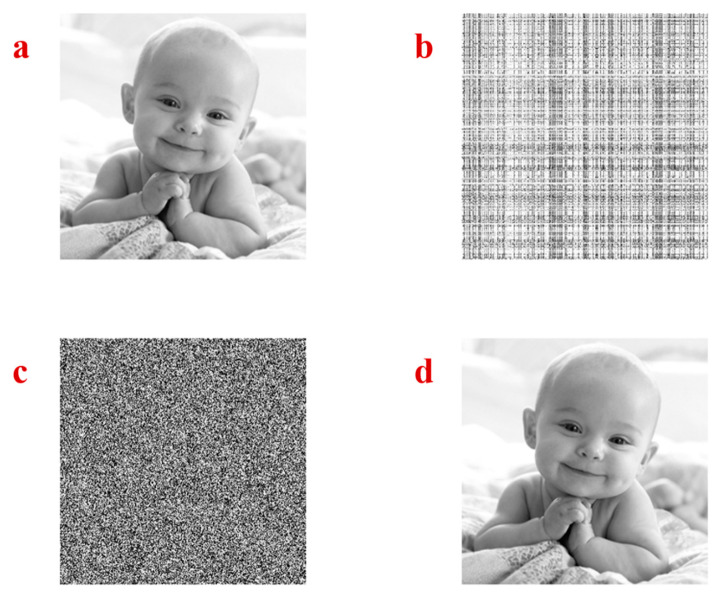
(**a**) The original baby image, (**b**) the shuffled rows and columns of the baby image, (**c**) the encrypted image, (**d**) the decrypted image.

As another example, the baby image is used (Figure 10a) to test the encryption method using the chaotic attractor of the proposed system. The shuffled rows and columns of the image using the random sequence generated by x and z variables are shown in Figure 10b. Then, the results are XORed with the random sequences which are generated by x and y variables and the encrypted image are shown in Figure 10c. The inverse process should be applied to decrypt the image on the receiver side, as shown in Figure 10d.

Altogether, the encryption algorithm is as follows:

**Algorithm 1** Encryption Algorithm1. Parameter and initial conditions are entered into the system.2. The proper run time’s step is selected.3. The float values of the time series are transferred to 32-bit binary values with 3 bits for the integer part and 29 bits for the fraction part. 4. The 20 least significant bits are selected to be used in the generation of random numbers, and they are put in a vector.5. The rows and columns are shuffled using the randomly generated indices from x and z variable of chaotic attractor.6. The random vectors generated by x and y of chaotic attractor are XORed, and then they are XORed with the shuffled image.7. The results are converted to decimal.

In the decryption, the inverse of the encryption steps is applied to the encrypted image. The results show that the decrypted images are loss-less since the mean square error of the original and decrypted image in both cases is zero. Dynamical properties of the previous section show that the system is highly dependent on initial conditions, which make the system a proper choice to be used in encryption. 

### 4.2. Encryption’s Performance

To investigate the performance of encryption, some measures are analyzed [55]. These measures help us to study the security of the encryption method. 

**Figure 11:**
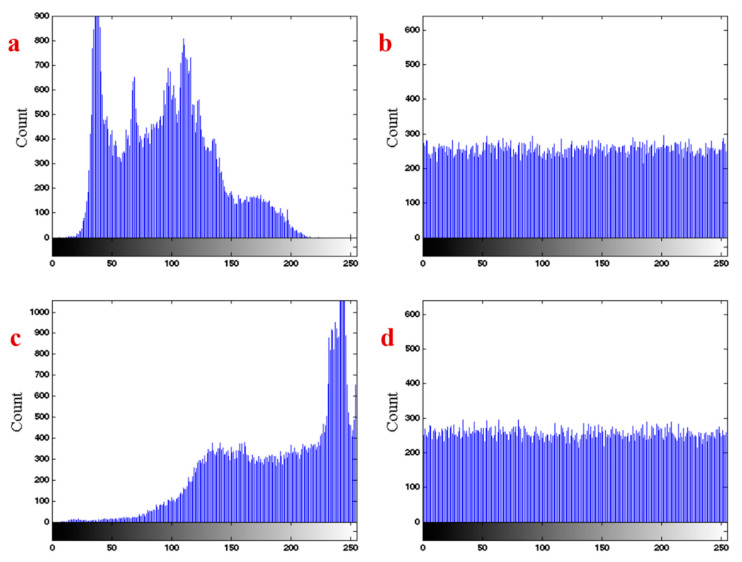
(**a**) The histogram of Lena image, (**b**) the histogram of the encrypted Lena image, (**c**) the histogram of the baby image, (**d**) the histogram of the encrypted baby image.

Histogram of an image shows its distribution of color values. Figure 11a shows the histogram of the Lena image. It can be seen that the distribution has a special form which depends on the colors of this figure. The desired encryption method should change the distribution of the encrypted image to uniform, so there is no possibility that the encrypted image can be broken. Figure 11b shows the histogram of the encrypted Lena image. It can be seen that the distribution is approximately uniform. Figure 11c,d shows the histogram of the original baby image and its encrypted image. The results present the power of the proposed chaotic system in the encryption. 

**Figure 12:**
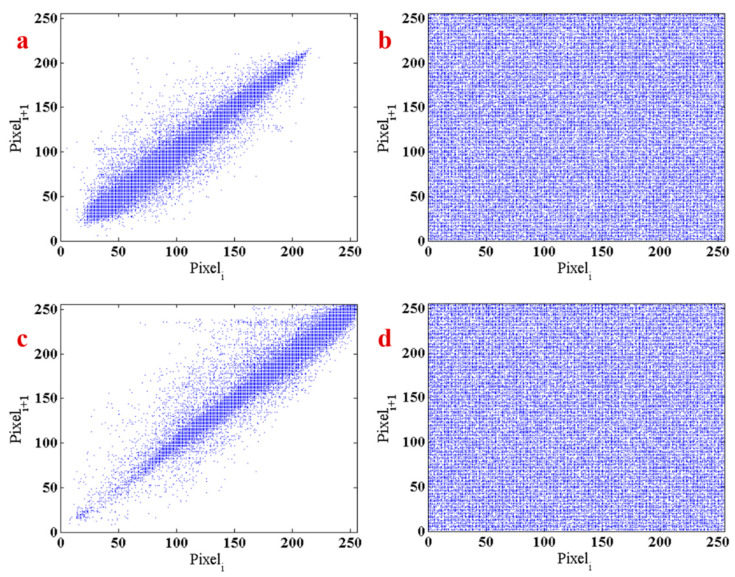
(**a**) The relation between pixels of Lena, (**b**) the correlation of the encrypted images of Lena, (**c**) the relationship between pixels of the baby image, (**d**) the correlation of the encrypted images of the baby.

The second measure is the correlation coefficient. This measure presents the relation between the pixels of the image. Figure 12a,c shows the relationship between the pixels of Lena and baby image, respectively. The correlation coefficient for these two images is 0.1633 and 0.0611. The graphic of the correlation of the encrypted images of Lena and baby are shown in Figure 12b,d. The results show that the encryption method using the proposed system has a proper correlation distribution. Correlation coefficients of the encrypted image are $-2.3585\times 10^{-4}$ and $1.5770\times 10^{-5}$, respectively. 

Another measure which is used in the calculation of quality of encryption method is Shannon entropy. Entropy calculates the complexity of the encrypted data. The calculated entropy of the original Lena and baby image is 7.2253 and 7.1606. This measure for encrypted Lena and the baby image is 7.9975, and 7.9974. The results show an increase in the entropy of the encrypted image. 

Encryption quality is another measure for the quality of encryption. This measure quantifies the difference of distribution of gray levels of the original and encrypted image. It is calculated as Equation (5). P and C are the original and encrypted image. P and C can have L gray levels as {0,…, L−1}. $H_{L}\left(P\right)$ is the number of occurrences of each gray level in the original image, and $H_{L}\left(C\right)$ is the number of occurrences of each gray level in the encrypted image [66]. In this study, both figures have 256 gray levels. This measure is $2.1734\times 10^{2}$ and $1.9934\times 10^{2}$ for Lena and baby image, respectively.

(5) $$EQ=\frac{\sum_{L=0}^{255}\left|H_{L}\left(C\right)-H_{L}\left(P\right)\right|}{256}$$

## 5. Conclusions

A new three-dimensional chaotic flow has been proposed in this paper. The system has a particular property in which its attractor should cross inside and outside of a determined cylinder. Equilibrium points and their stabilities were analyzed in this paper. Bifurcation diagram of the system was studied using backward continuation method and constant initial conditions method. The results show that the system has multistability. Lyapunov exponents of the system were studied to determine the chaotic regions with respect to changing the bifurcation parameter. Entropy analysis of the system was used to investigate the complexity of attractors by changing the parameter. Finally, the proposed system was applied in an Algorithm 1, and its performance was discussed. Some Matlab codes of the paper are available in the following link, https://drive.google.com/drive/folders/1y96B_VZb1qQ7FLuNDiVEHZoG6K9gJT76?usp=sharing.
